# Functional Reconstruction of Thumb with Tendons from an Amputated Finger in Median Nerve Territory-Oriented Macrodactyly

**DOI:** 10.1055/s-0045-1813227

**Published:** 2025-12-24

**Authors:** Shanmuganathan Raja Sabapathy, Monusha Mohan, Gugri Manjunatha Sunay, Shruthi Chandrasekar

**Affiliations:** 1 Department of Plastic, Hand and Reconstructive Microsurgery, Ganga Hospital, Coimbatore, Tamil Nadu, India

**Keywords:** macrodactyly, nerve territory-oriented macrodactyly, opponensplasty

## Abstract

Nerve territory-oriented macrodactyly (NTOM) of the hand, particularly involving the median nerve, results in severe enlargement and dysfunction of the thumb and index finger, compromising grasp and pinch functions. We present two paediatric cases managed by ray amputation of the index finger and debulking of the thumb, with a novel approach utilizing tendons from the amputated finger to restore thumb flexion, abduction, and opposition in a single-stage procedure. This technique avoids additional donor-site morbidity and optimizes the use of available structures. Both patients achieved improved hand function and aesthetics, with one attaining near-normal pinch and grip strength and the other achieving early pinch ability postoperatively. Our experience suggests that preoperative planning for the use of these tendons is crucial and that careful intraoperative identification enhances outcomes. This method offers a new reconstructive option for NTOM, emphasizing both functional and cosmetic restoration in this challenging congenital condition.

## Introduction

Nerve territory-oriented macrodactyly (NTOM), when it involves the median nerve, causes severe gigantism of the thumb and index finger. There is extensive involvement of the palmar side compared to the dorsum, causing hyperextension deformities at the metacarpophalangeal (MCP) and interphalangeal (IP) joints. Lipomatous infiltration of the thenar muscles affects thumb opposition. The gigantic digits hinder the function of the ulnar three fingers. Eventually, the hand is devoid of pinch and grasp functions.

Management involves ray amputation of the index finger, debulking of the thumb, and secondary surgeries to improve thumb position and movement. We describe two patients in whom tendons from the amputated index were used to restore flexion, abduction, and opposition of the thumb in a single operation. To our knowledge, this primary reconstruction in NTOM using amputated finger tendons has not been previously reported.

## Case Report

### Patient 1

**Fig. 1:**
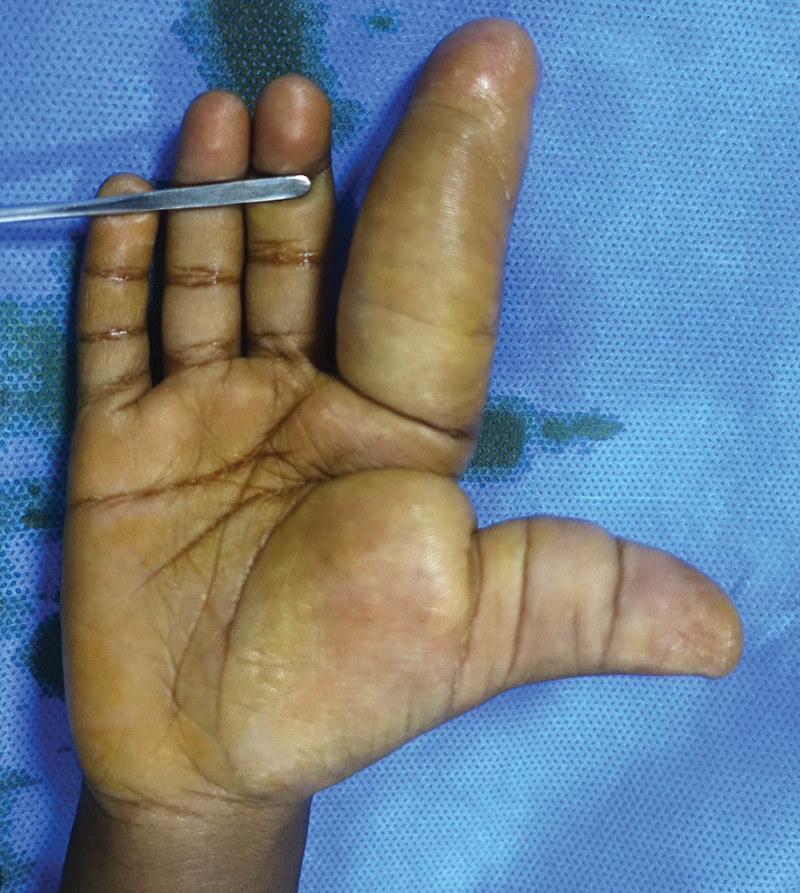
A 4-year-old boy with a gigantic immobile thumb and index finger. The thumb lies away from the palm with absent abduction and opposition. Thenar hypertrophy was present.

A 4-year-old boy was brought to us with an enlarged, immobile thumb and index finger ( Fig. 1 ). He used only the ulnar three digits for grasping. Amputation of the index finger and debulking of the thumb and palm were planned.

**Fig. 2:**
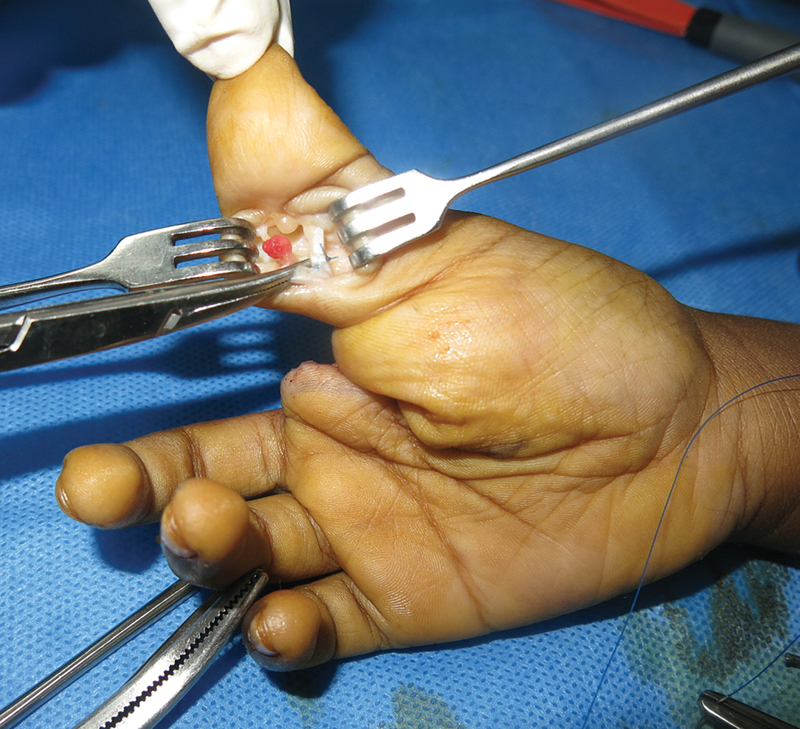
Intraoperative photograph after ray amputation of the index finger. Augmentation of flexion was done by attaching the FDP and FDS tendons of the index finger to the FPL tendon. FDP, flexor digitorum profundus; FDS, flexor digitorum superficialis; FPL, flexor pollicis longus.

**Fig. 3:**
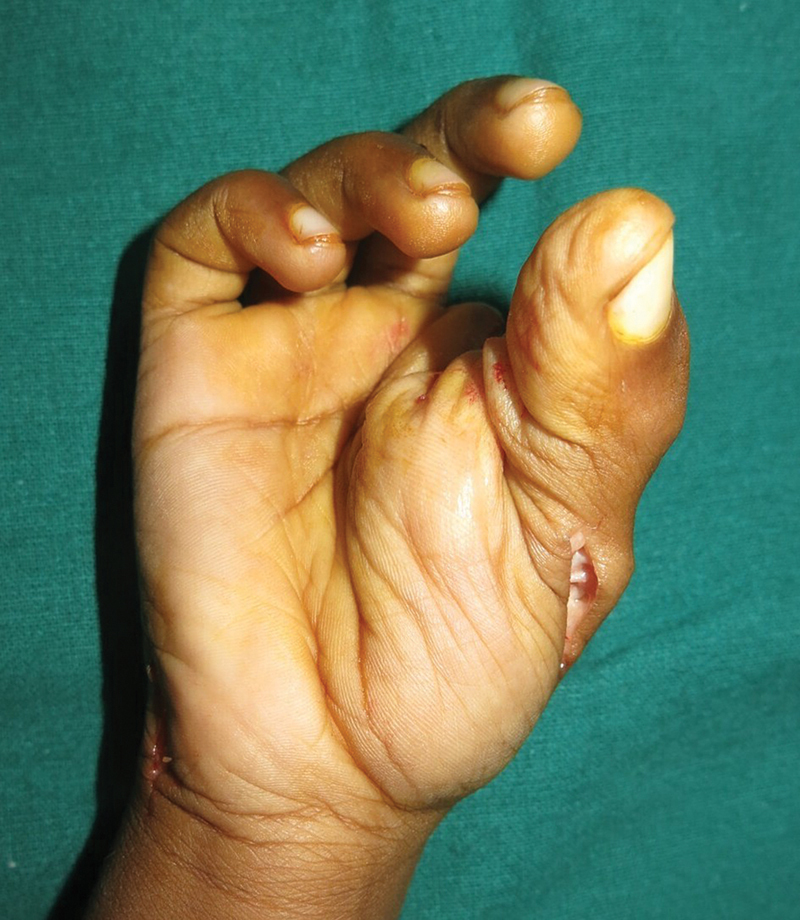
The posture of the hand at completion of debulking and EIP opponensplasty. EIP, extensor indicis proprius.

Intraoperatively, the index flexors and extensors had good excursion. We used these tendons to restore thumb movement by dividing them distally. After debulking the thumb and the thenar region, both flexors were dissected to the level of the carpal tunnel through the same incision. The flexor digitorum profundus (FDP) was woven into the flexor pollicis longus (FPL), correcting extension deformity ( Fig. 2 ). The flexor digitorum superficialis (FDS) was also sutured to FPL for added flexion. The extensor indicis proprius (EIP) was rerouted and transferred to the abductor pollicis brevis for opposition ( Fig. 3 ). The detached dorsal interosseous was transferred to the radial lateral band of the middle finger extensor. The hand was immobilized in a cast for 3 weeks, then a thermoplastic splint for 3 more weeks, followed by night splinting for another 3 weeks.

**Fig. 4:**
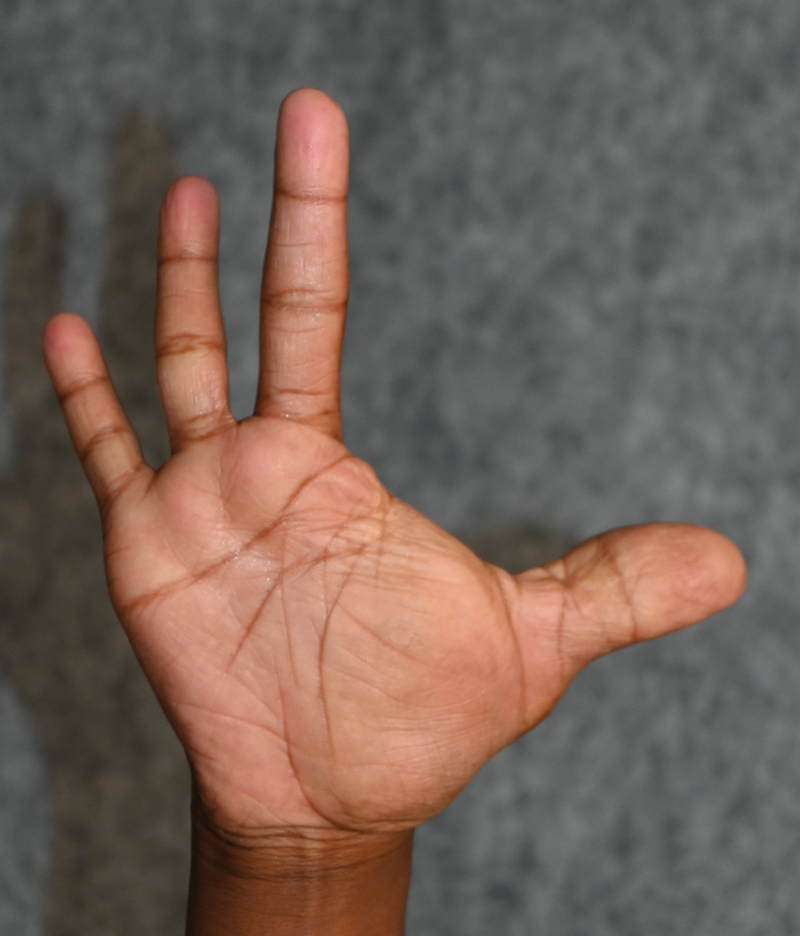
Clinical image of the operated hand at 3-year follow-up.

**Fig. 5:**
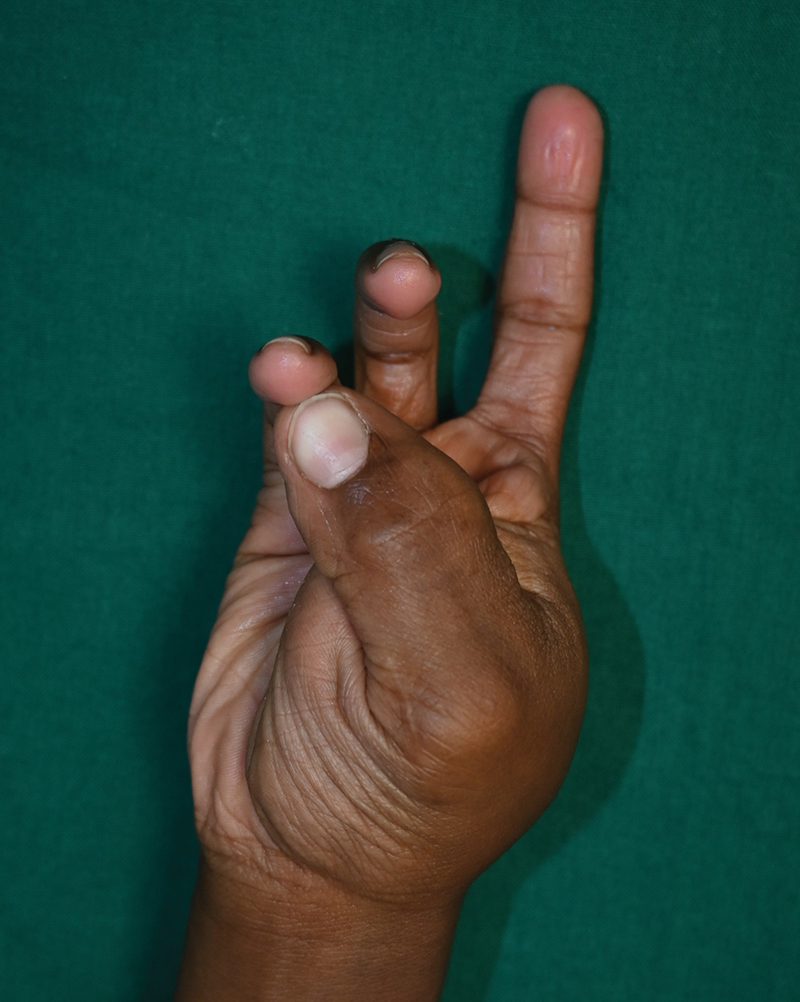
Excellent opposition at final follow-up.

**Fig. 6:**
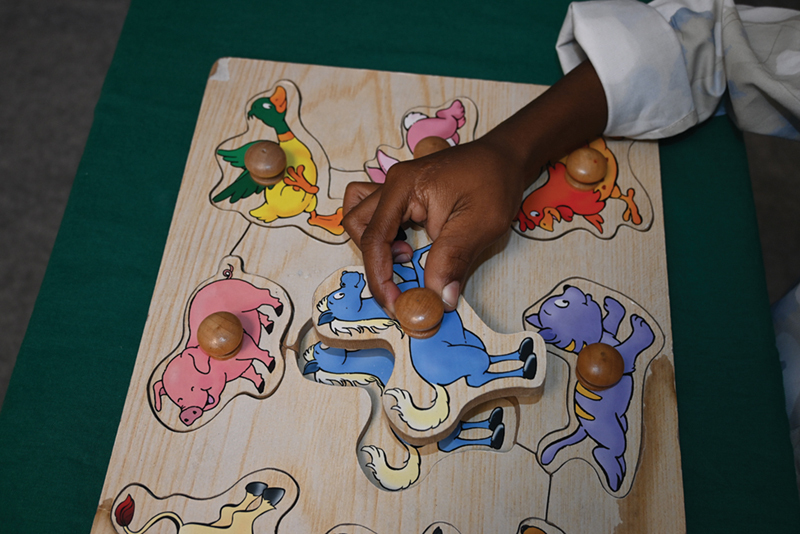
Good pinch activity after the reconstructive surgery.

At the 3-year follow-up, the child's hand was aesthetically and functionally good ( Figs. 4 , 5 , 6 ). He achieved thumb–middle finger pinch, with pulp-to-pulp and tripod pinch strengths of 1.5 and 1.3 kg (same as normal). The lateral pinch was 1.8 kg (normal: 2.2 kg), and the grip strength was 6.7 kg (normal: 8 kg). Thumb opposition was excellent (Kapandji score: 8). The first web angle was 80°. The range of motion (ROM) at the thumb MCP joint was 0 to 65° and at the IP joint it was 0 to 15°. Sensations were mildly diminished (two-point discrimination: 8 mm).

The parent-proxy Patient-Reported Outcomes Measurement Information System (PROMIS) questionnaire (Pediatric Upper Extremity-Short Form 8a) showed that the child could button his shirt or pants, open a jar, open the rings in school binders, pour a drink from a full pitcher, pull a shirt over his head, pull open heavy doors, put on his shoes, and use a key to unlock a door, all by himself, with no difficulty. Parents rated function and appearance as 10/10 compared to 1/10 before surgery.

### Patient 2

**Fig. 7:**
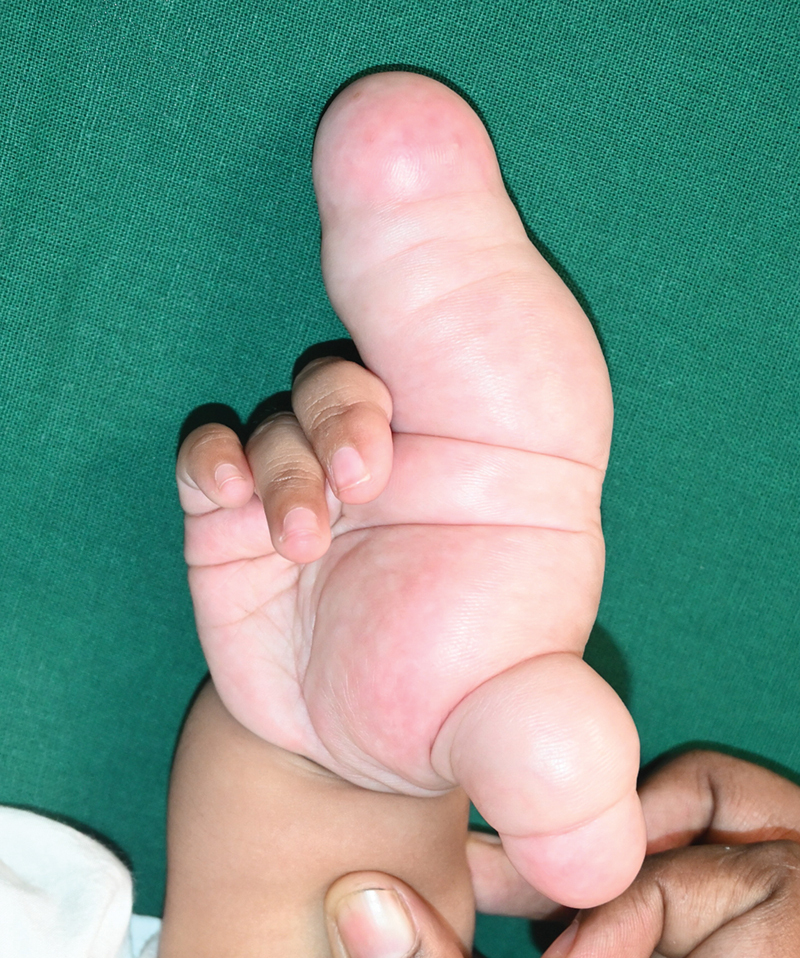
Clinical image of the macrodactylous hand of the second child.

**Fig. 8:**
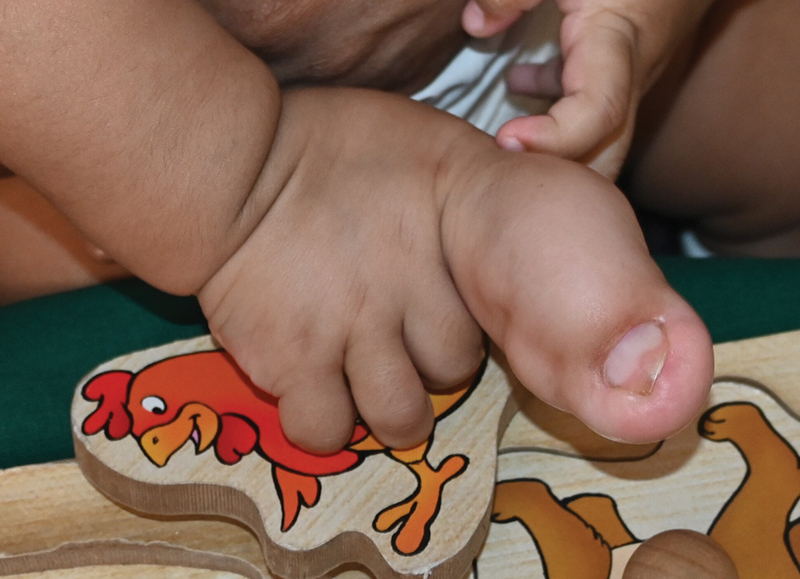
Immobile macrodactylous thumb and index finger. Healthy ulnar three fingers used for grasping.

A 6-month-old girl was born with an enlarged, immobile thumb and index finger ( Figs. 7 and 8 ). The index finger was amputated and its FDP was used for opponensplasty. The FPL was plicated to augment flexion. We did not use EIP due to the problem of reach.

At 2 months, the ROM at the thumb MCP joint was 0 to 65° (passive) and the IP joint was in 25° of hyperextension. The first web angle was 55°. The mother rated the function and appearance as 7/10 (preoperative: 6/10).

## Discussion

Achieving acceptable aesthetics and function in macrodactyly is a challenge, which prompted Flatt to say that “Macrodactyly dwarfs the giants in hand surgery.” In median NTOM, the goal is to obtain unfettered action of the uninvolved fingers and position the thumb in a place where the functional fingers could meet it to make pinch and grasp possible.

The first goal is obtained by ray amputation of the index finger. The second goal of making the thumb functional is more challenging. First, the bulk by itself prevents movement. Second, the thenar muscles are functionally compromised due to severe lipofibromatous infiltration, which makes debulking without damaging the muscles difficult. They might also be atrophied due to median nerve compression at the carpal tunnel. 1 Additional power to the thumb will be helpful.

Tendons of the amputated index are a great resource. Their use must be planned in advance so that they can be used; this thought must be present prior to operation, since the tendons have to be harvested as distally as possible. In both the children, we found that they had good excursion on proximal traction but were ineffective due to severe hypertrophy and secondary changes in the joint. One notable feature is that the flexors were found very displaced into the ulnar gutter at the MCP joint and we recommend diligent exploration and prior isolation before amputation. This becomes all the more important if the tendons are not hypertrophied, as in our cases.

Two issues must be addressed: augmenting flexion and enabling opposition. The first can be achieved by plicating the FPL and using an index flexor. Opposition can be achieved by using the index flexor or extensor. We used the index EIP and FDP for our cases, respectively. The index FDP acts independently and will not affect the other fingers. Good tension compensates for the possible stretching over time. Even if movement is limited, correct thumb positioning enhances function.

Only a few reports describe opponensplasty for reanimating the thumb. 1 2 3 Rousso et al used the extensor pollicis brevis tendon for an enlarged, stiff thumb. 1 The others used the palmaris longus and FDS of the middle finger respectively for thenar wasting. 2 3 By using tendons from the amputated finger, we avoid additional donor-site morbidity and retain options for future procedures. This technique combination has not previously been reported.

The timing of surgery should be individualized, depending on surgeon's preference and the child's health. Debulking can be performed at any age. Since tendon transfers require tension adjustment and rehabilitation, we recommend waiting until after the first year. Surgical outcomes may be influenced by bone and joint deformities developing over time. 1 4 5

Functional restoration is as important as cosmesis in hand macrodactyly. Assessing hand function and identifying problems are key to surgical planning. Opponensplasty, if required, should be performed early along with debulking. In this report, we could utilize the amputated index finger tendons for transposition.

The authors have no sources of financial or material funding to disclose. No portion of this work has been previously presented or published. All the authors were involved in the clinical care of this patient, the design and conception of this paper, and the writing and revising process. This study was exempt from institutional board review per our institutional policy on small case reports. Our study was performed in accordance with and conforming to the Declaration of Helsinki.
